# Does vitamin D supplementation improve bone health, body composition and physical performance beyond endurance exercise in patients with type 2 diabetes: A secondary analysis of randomized controlled trial

**DOI:** 10.3389/fphys.2022.1003572

**Published:** 2022-09-28

**Authors:** Xiaomin Sun, Wenjuan Xiao, Zhongying Li, Sirui Zhou, Mengyue Dong, Cong Huang, Yan Ma, Bo Gou

**Affiliations:** ^1^ Global Health Institute, School of Public Health, Xi’an Jiaotong University Health Science Center, Xi’an, China; ^2^ Rehabilitation Center, Beijing Rehabilitation Hospital of Capital Medical University, Beijing, China; ^3^ Department of Health Science, Xi’an Physical Education University, Xi’an, China; ^4^ Department of Sports and Exercise Science, Zhejiang University, Hangzhou, China

**Keywords:** vitamin D supplementation, exercise, bone health, body fat, physical performance

## Abstract

This study aimed to assess the effects of a 12-week vitamin D and endurance exercise intervention on bone health, body composition and physical performance among patients with type 2 diabetes. Totally, 61 patients were randomly assigned to vitamin D (VDG), exercise (EG), vitamin D and exercise intervention (VEG), and control (CG) groups. Bone health (bone mineral density, BMD; bone mineral content, BMC), body composition and physical performance were measured before and after the intervention. Dual energy X-ray absorptiometry was used to assess bone health and body composition. There were no additive effects of vitamin D beyond exercise were observed. Vitamin D supplementation had significant effects on maintaining bone health compared with their counterpart Total (BMC, EG + CG: 2,719.9 ± 70.0 vs. 2,670.1 ± 65.6; VDG + VEG: 2,610.9 ± 88.2 vs. 2,605.3 ± 84.8; trunk BMC, 870.2 ± 26.8 vs. 836.3 ± 23.7; 824.8 ± 29.5 vs. 822.1 ± 27.8; spine BMD, 1.15 ± 0.03 vs. 1.11 ± 0.02; 1.09 ± 0.03 vs. 1.09 ± 0.02) were observed. Exercise had a main effect on the reduction of total and trunk BF%. Patients in EG had a decreased BMC, while it was alleviated in VEG after intervention. Although no additive effect of vitamin D supplementation beyond exercise training, the supplementation had a potential effect on the prevention of bone loss induced by exercise only.

## Introduction

With rapid social and economic developments during the past 3 decades, China is facing a growing threat from non-communicable chronic diseases (NCDs), and diabetes is considered one of the most common NCDs in China and several other countries (GBD 2013 Mortality and Causes of Death Collaborators, 2015). The most recent national representative surveys reveal that diabetes prevalence among Chinese adults has increased from 10.9% in 2013 to 12.4% in 2018, respectively (Wang et al., 2021). Poorly controlled high blood glucose can lead to serious consequences, including increased risk of heart disease and stroke (Forbes and Cooper, 2013; Harding et al., 2019).

Some studies reported that diabetes is associated with lower bone mineral density (BMD) levels and the rapid loss of skeletal muscle strength and quality (Park et al., 2006; Park et al., 2007; Zhou et al., 2010; Wong et al., 2013), and all of these complications may induce physical impairment. Although both endurance and resistance exercise can improve insulin actions and glucose control, moderate endurance exercise is considered as the first choice for patients with T2DM. Accumulating evidence reported that endurance exercise is more likely to be associated with greater BMI reduction, accompanying with greater effect in the improvement of glucose control than resistance exercise (Yang et al., 2014). Prior evidence indicates exercise following weight loss may contribute to high rates of bone loss in patients with T2DM (Lipkin et al., 2014; Beavers et al., 2017), which may due to high bone turnover (Hansen et al., 1991; Ravn et al., 1999). Thus, optimizing T2DM management not to have a negative effect on bone health and physical performance has become a necessity in T2DM treatment.

It is generally agreed that vitamin D has a classic role in the maintaining normal levels of serum Ca and P, thus increased serum 25-hydroxyvitamin D (25(OH)D) was reported to have beneficial effect on osteoblast (Zhang et al., 2021). Additionally, the associations of low serum 25(OH)D levels with higher risk of decreased muscle mass and impaired physical performance were corroborated in cross-sectional or longitudinal studies (Bischoff-Ferrari et al., 2004; Wicherts et al., 2007; Laird et al., 2010; Houston et al., 2012; Halfon et al., 2015; Shardell et al., 2015; Aspell et al., 2019).

Recent studies shown that exercise may also be involved in the regulation of vitamin D. Prior evidence reported that exercise significantly increased vitamin D receptor (VDR) expression (Makanae et al., 2015; Aly et al., 2016), which plays a key role in the regulation of vitamin D actions. Consistently, our recent studies also observed that endurance exercise training could increase serum 25(OH)D concentrations or prevent its seasonal reduction in young and old men (Sun et al., 2017; Sun et al., 2018). Additionally, vitamin D supplementation could directly upregulate the AMPK-GLUT-4 signaling pathway through VDR related (phosphorylated ERK1/2 and Mnk1) expression to increase glucose utilization and participate in exercise pathways of glucose utilization pathways (Makanae et al., 2015). Considering a substantial impact of vitamin D in bone and musculoskeletal system, co-exposure to vitamin D beyond exercise may provide additional benefits on bone health and physical performance in patients with T2DM via VDR pathway (Park et al., 2007). Our previous works reported that higher levels of cardiorespiratory fitness (raised by endurance exercise training) and serum 25(OH)D concentrations were associated with a greater favorable glucose homeostasis (i.e., lower fasting insulin and insulin resistance) than alone in middle-aged and elderly men (Zhou et al., 2010). However, few randomized controlled studies has conducted to assess the potential additive effects of endurance exercise combined with vitamin D intervention on bone health, body composition and physical performance in patients with T2DM.

This study aimed to investigate effects of a 12-week endurance exercise training with or without vitamin D supplementation on bone health, body composition and physical performance among patients with T2DM. Our study would provide a comprehensive exploration on bone and musculoskeletal health during T2DM treatment.

## Methods

### Trial design

The study was a 12-week, randomized, placebo-controlled vitamin D and exercise intervention trial with four arms. T2DM was diagnosed according to the 1999 World Health Organization (1999) guidelines on diabetes. The patients were eligible if they meet the following inclusion criteria: 1) diagnosed with T2DM ≤ 10 years without insulin therapy; 2) without regular vitamin D and/or calcium supplements in the past year; 3) without regular exercise habits in the past year; 4) without renal insufficiency, osteoporosis and fracture; and 5) without sunlight exposure history. They were also excluded if they experienced acute infection and complication of diabetes, or had metal implants in the body that could affect magnetic resonance imaging and dual-energy X-ray absorptiometry (DXA) measurements.

To ensure a randomized, double-blind (for vitamin D supplementation) effect, trial designers, testers, and data collectors were blinded to the vitamin D/placebo intake grouping until the intervention trial and data collection were completed. All patients were instructed not to perform any formal exercise or change their general physical activity levels and dietary habits during the interventional period. Participants were asked to abstain from caffeine, alcohol, tobacco, and strenuous physical activity before blood sample collection, and all measurements were assessed at baseline and at the end of the intervention.

**FIGURE 1:**
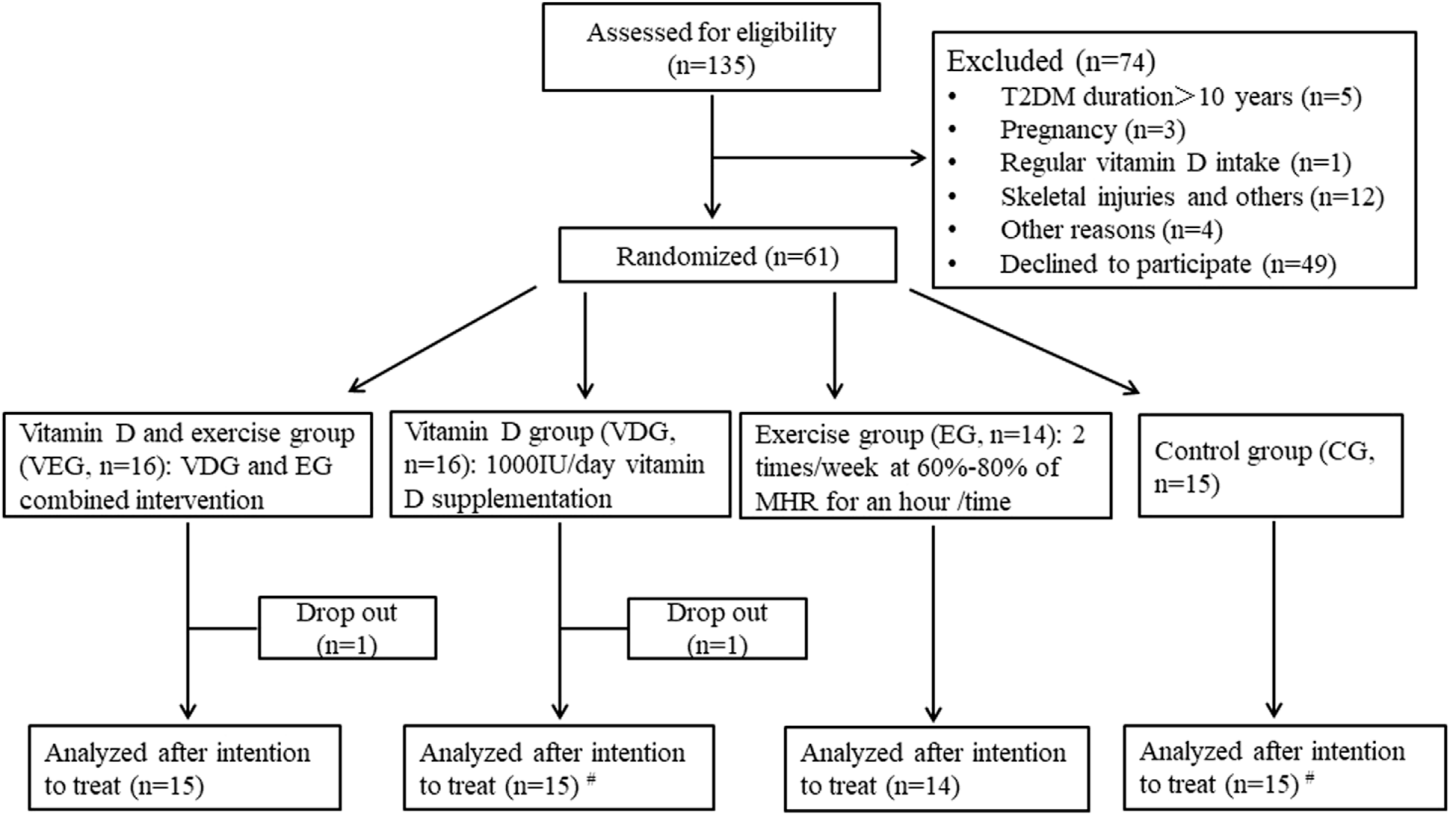
Flow diagram of participants. ^#^One participant in the vitamin D supplementation group had incomplete dual-energy X-ray absorptiometry and physical performance measurements, and another in the control group did had incomplete dual-energy X-ray absorptiometry measurements. MHR, maximum heart rate.

The study was approved by the Xi’an Jiaotong University Health Science Center and in accordance with the approved corresponding ethical guidelines. All participants signed an informed consent form after they were informed of the purpose, procedures, and risks of this study. The study was registered in the Chinese Clinical Trial System (registration number: ChiCTR1800015383). Details of flow diagram of patients were presented in Figure 1.

### Setting and participants

A total of 61 patients were randomly allocated to one of the four groups using a computer-generated list with a random allocation sequence. The groups were: 1) vitamin D supplementation (VDG: N = 16); 2) exercise training (EG: N = 14); 3) vitamin D combined with exercise (VEG: N = 16); and 4) control group (CG: N = 15).

Patients in the VEG and VDG were allowed to take a daily vitamin D supplementation of 1,000 IU (Nature Made, Otsuka Pharmaceutical Co, Ltd, Tokyo, Japan), while those in the EG and CG received a placebo tablet identical to vitamin D supplements in appearance, shape, and color. The placebo tablets contained only starch, cellulose, and magnesium stearate. All supplements were prepared in identical bottles and were sent or hand-delivered to the home of patients monthly, and participants were asked to remotely report weekly. Subjects who did not take the tablet for seven consecutive days were excluded. The dose of vitamin D supplements was elected based on the Institute of Medicine guideline and prior evidence (Ross et al., 2011; Wu et al., 2017).

Patients in the VEG and EG attended supervised, progressive cycling training classes for an hour at least 2 times/week for 12 weeks, with 60%–80% of their maximum heart rate (HRmax) (Supplementary Table S1). The exercise frequency was recorded. HRmax was calculated using the following formula: HRmax=(220-age) (Tanaka et al., 2001). During the exercise, rating of perceived exertion (RPE) and polar monitors were used to monitor the heart rate, and compliance with each protocol was recorded. The classes were supervised by a qualified trainer who was knowledgeable about the protocol and procedures of this study. Participants attended a 5-min warm up on a treadmill at 50%–60% of their HR-max and subsequently followed the 50-min endurance exercise protocol (mainly cycling) assigned to them combined with a 5 min recovery exercise at 40%–50% of their HR-max comprising cycling and stretching.

### Anthropometric and body composition measurement

Height and body mass were measured with the participants wearing light clothing and being barefoot, and the body mass index (BMI, kg/m^2^) was calculated by dividing the body mass (kg) by the square of height (m^2^). Body muscles, trunk muscles, body fat percentage (BF%) and trunk body fat percentage (TBF%), bone mineral content (BMC), and BMD were estimated using DXA (Hologic QDR-4500, DXA Scanner, Hologic Inc, Waltham, MA, United States) by a recognized technologist.

### Physical performance

Grip strength (kg) was measured using a hand-grip dynamometer (HK6800-WL, China). Patients held the dynamometer with the arm completely extended while standing and exerted their maximal grip force with each contraction trial. This process was repeated for both dominant and non-dominant hands, and the highest value of each hand was recorded. The mean value of the two maximal grip strength scores was used.

Response latency was defined as the elapsed time (second, s) between the delivery of the electrical stimulus and the subject response using a reaction tester (HK6800-FY, China). Expended response time indicated that the body responds to a stimulus slowly. The single leg balance test was measured to the nearest 0.001 s with patients naturally standing on one foot with their eyes closed for two tests; the best test result was selected. Vital capacity was defined as the maximum amount of air that a subject can expel from the lungs after maximum inhalation using a spirometer (GMCS-II, China).

### Physical activity

The International Physical Activity Questionnaire (IPAQ) was used to assess the physical activity of patients, except for when they were attending the exercise program. The IPAQ includes a comprehensive range of physical activities and refers to activities 1 week before completing this questionnaire. The questionnaire was reported to be appropriate for individuals aged ≥18 years and is suitable for the Chinese population (Craig et al., 2003). The level of physical activity that the respondents were engaged in each week was calculated as follows: the corresponding level of individual physical activity = the Metabolic Equivalent assignment × weekly frequency (day/week) × time of day (min/day).

### Serum 25(OH)D concentrations

After overnight fasting for 12 h, blood samples were collected from the antecubital vein of the forearm between 8:30 a.m. and 10:00 a.m. by well-trained nurses at baseline (0-week), at 6-week and 12-week. Serum samples were transferred to separate tubes and frozen immediately at −80°C. Serum 25(OH)D concentrations were quantified using electrochemiluminescence immunoassays (Roche Diagnostics GmbH, Mannheim, Germany) at three points. Intra- and inter-assay variation coefficients for 25(OH)D were 1.96% and 3.78%, respectively.

### Sunlight exposure score

A self-reported questionnaire with good reliability and validity was used to evaluate the duration of sunshine exposure in a week before and after intervention. The mean weekly sunlight exposure score was calculated. Additional details were provided elsewhere (Hanwell et al., 2010).

### Statistical analysis

Descriptive statistics were calculated as mean ± standard deviation for continuous variables and numbers (percentages) for categorical variables at 95% confidence intervals (CI). The Shapiro-Wilk test was performed to assess data distribution normality, and non-normally distributed data were normalized by logarithmic transformation (single leg balance test). To compare baseline characteristics among groups, a one-way analysis of variance was used for continuous variables, whereas chi-squared test and fisher’s exact test were used to categorical variables. A factorial repeated measures ANOVA was used to determine whether there were any major (vitamin D or exercise) or interaction (vitamin D × exercise) effects among groups. A post hoc test using Bonferroni correction was utilized to identify any significant differences between groups when a significant main or interaction effect was identified. An intention-to-treat analysis was performed for all data using SPSS version 22.0 software (SPSS, Inc, Chicago, IL, United States). A *p*-value <0.05 was considered statistically significant.

This study reports the secondary outcomes of a registered randomized, double-blind (for vitamin D supplementation), placebo-controlled clinical trial (registration number: ChiCTR1800015383). Power calculations indicated that a sample size of 12 participants in each group will have a statistical power of 85% and an effect size of 0.26 for examining the association between vitamin D intervention and homeostasis model assessment insulin resistance (Sun et al., 2016). The sample size was expanded to 60 participants to account for a 20% loss to follow-up. Ultimately, 61 participants were enrolled in the study. Power calculations were performed using G*Power software version 3.1.9.2 (Faul et al., 2007).

## Results

### Baseline characteristics

One patient in VDG and another in VEG withdrew from the study due to personal reasons during the intervention; two did not complete DXA and one failed for physical performance measurements due to time conflicts (Figure 1). No trial-related adverse event was reported during the intervention. Finally, 59 patients completed the 12 weeks intervention. Of 29 patients in exercise groups (VEG + EG), 9 exercised 3 times/week, 11 for 2–3 times/week, and 6 exercised 1–2 times/week.

Table 1 present the baseline characteristics of the 59 patients in this study. The mean age was 50.1 ± 7.3 years, and mean BMI was 25.9 ± 3.6 kg/m^2^. No significant differences were observed in age, gender (%), BMI, BF%, physical activity, and Sun exposure score between groups. Of the 59 patients, 39 (66.1%) had vitamin D deficiency (25(OH)D levels <20 ng/ml), and 18 (30.5%) had vitamin D insufficiency (25(OH)D levels: 20–30 ng/ml) at baseline.

**TABLE 1:** Subject characteristics at baseline in 59 patients with type 2 diabetes mellitus at baseline.

Variables	Overall	VEG	VDG	EG	CG	P
	n = 59	n = 15	n = 15	n = 14	n = 15	
Age (years)	50.1 ± 7.3	50.6 ± 6.6	51.2 ± 7.2	47.9 ± 8.6	50.6 ± 7.0	0.620
Men (%)	44 (71.2%)	10 (60.0%)	12 (73.3%)	10 (71.4%)	12 (80.0%)	0.711
Height (cm)	167.4 ± 7.6	165.1 ± 8.1	167.6 ± 8.5	169.1 ± 7.7	167.8 ± 6.1	0.550
Weight (kg)	72.9 ± 13.1	68.9 ± 13.1	71.4 ± 13.1	74.7 ± 15.1	76.9 ± 10.9	0.364
BMI (kg/m^2^)	25.9 ± 3.6	25.1 ± 3.0	25.3 ± 3.3	26.1 ± 4.8	27.2 ± 3.2	0.363
Body fat (%)^a^	30.3 ± 7.7	32.0 ± 6.1	27.9 ± 9.2	30.9 ± 8.6	30.3 ± 6.6	0.531
Physical activity (MET-min/Week)	3,578 ± 3,454	3,164 ± 2,413	4,065 ± 4,583	4,334 ± 4,286	2,801 ± 1976	0.596
Sun exposure score	14.8 ± 7.3	16.5 ± 8.3	13.6 ± 4.1	16.1 ± 8.8	13.2 ± 7.3	0.506
Diabetes duration (years)	3.5 ± 2.4	3.3 ± 2.4	3.9 ± 2.4	3.1 ± 2.5	3.7 ± 2.6	0.856

a Data are mean ± standard deviation or n (%). VEG, vitamin D combined exercise intervention group; VDG, vitamin D intervention group; EG, exercise intervention group; CG, control group; BMI, body mass index; MET, metabolic equivalent. Data were analyzed by one-way ANOVA, or chi-square test, n = 14 in CG, were included. Boldface indicates statistically significance.

### Serum 25(OH)D concentrations

**FIGURE 2:**
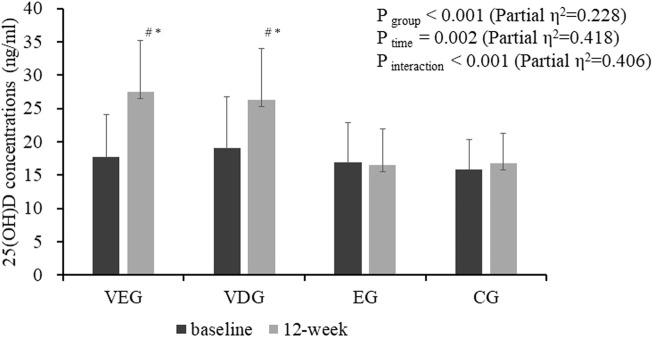
Effect of vitamin D supplementation on serum 25(OH)D concentrations in 59 patients with type 2 diabetes mellitus. Values are expressed as mean ± standard deviation. VEG, vitamin D combined exercise intervention group; VDG, vitamin D intervention group; EG, exercise intervention group; CG, control group. Two way repeated-measures ANOVA was used to determine the effect of time and group on serum 25(OH)D. ^#^, *p* < 0.05 vs. the baseline within group; *, *p* < 0.01 vs. exercise or control group at the same time point.

After vitamin D supplementation for 12 weeks, serum 25(OH)D concentrations significantly increased in VDG and VEG compared with those at baseline, respectively (VEG: 17.7 ± 6.4 ng/ml vs. 27.5 ± 7.8 ng/ml; VDG:19.0 ± 7.7 ng/ml vs. 26.3 ± 7.7 ng/ml, all *p* < 0.05); however, there were no significant changes were observed in EG and CG groups. At the end of the intervention, serum 25(OH)D concentrations were higher in VEG and VDG than in EG and CG (*p* < 0.05); no difference was observed between VDG and VEG at any time points (Figure 2). Moreover, vitamin D deficiency rate significantly decreased from 66.7 to 20.0% in the vitamin D (+) group and increased from 65.5 to 72.4% in the vitamin D (-) group.

### Body composition and bone health

**FIGURE 3:**
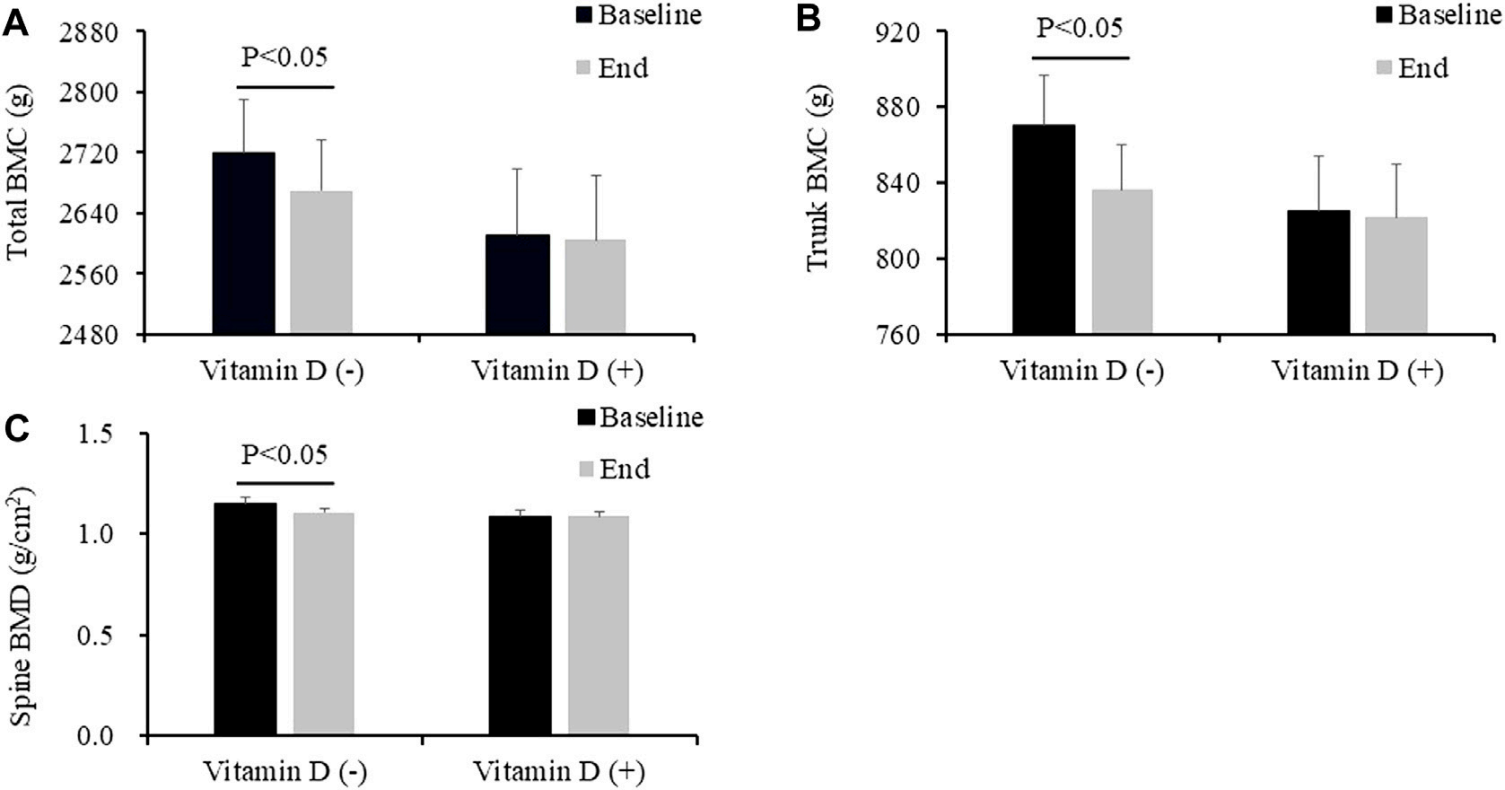
Effect of vitamin D supplementation on total body BMC **(A)**, trunk BMC **(B)**, and Spine BMD **(C)**, according to the vitamin D treatment group. Values are expressed as mean ± standard error. VEG, vitamin D combined exercise intervention group; VDG, vitamin D intervention group; EG, exercise intervention group; CG, control group; BMC, bone mineral content; BMD, bone mineral density. Vitamin D (+), VEG + VDG; Vitamin D (-), EG + CG.

**FIGURE 4:**
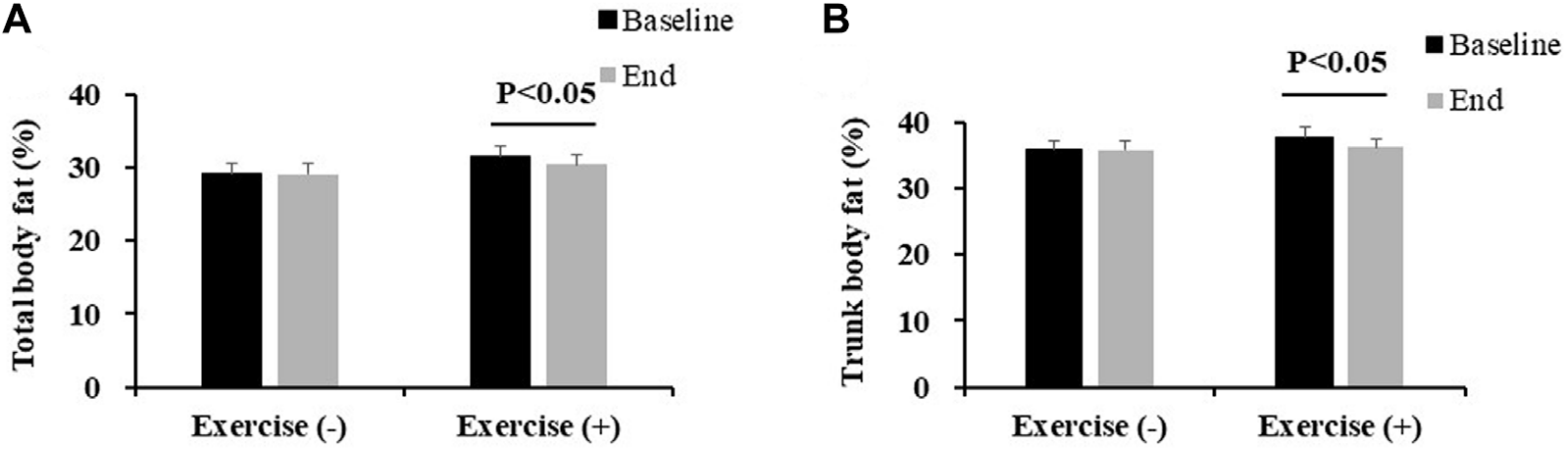
Effect of exercise training on body fat percentage **(A)** and trunk body fat percentage **(B)**, according to the exercise intervention group. Values are expressed as mean ± standard error. VEG, vitamin D combined exercise intervention group; VDG, vitamin D intervention group; EG, exercise intervention group; CG, control group; BMC, bone mineral content; BMD, bone mineral density. Exercise (+), VEG + VDG; Exercise (-), EG + CG.

As shown in Table 2, there were no additive effects of vitamin D beyond exercise on any bone health, body composition and physical performance variables. Significant vitamin D effects were noted in the total BMC (*p* = 0.014, partial η^2^ = 0.109), trunk BMC (*p* = 0.046, partial η^2^ = 0.073), spine BMD (*p* = 0.046, partial η^2^ = 0.073), and significant exercise main effect was noted in the total BF% (*p* = 0.025, partial η^2^ = 0.092) and trunk BF% (*p* = 0.032, partial η^2^ = 0.084). The post hoc test shows vitamin D supplementation had significant and slight effects on maintaining bone health (total BMC, baseline vs. end, EG + CG: 2,719.9 ± 70.0 vs. 2,670.1 ± 65.6; VDG + VEG: 2,610.9 ± 88.2 vs. 2,605.3 ± 84.8; trunk BMC, 870.2 ± 26.8 vs. 836.3 ± 23.7; 824.8 ± 29.5 vs. 822.1 ± 27.8; spine BMD: 1.15 ± 0.03 vs. 1.11 ± 0.02; 1.09 ± 0.03 vs. 1.09 ± 0.02); exercise could significantly decrease total body fat%) VDG + CG, 29.2 ± 1.5 vs. 29.1 ± 1.4; VEG + EG, 31.5 ± 1.4 vs. 30.4 ± 1.4; trunk body fat%: 35.8 ± 1.5 vs. 35.8 ± 1.5; 37.7 ± 1.5 vs. 36.2 ± 1.4) (Figures 3, 4).

**TABLE 2:** The parameters of body composition, bone health and physical performance by four groups at baseline and at end.

Variables	VEG N = 15	VDG N = 14	EG N = 14	CG N = 15	Vitamin D effect		Exercise effect		Interaction effect	
					P	Partial η^2^	P	Partial η^2^	P	Partial η^2^
**Body composition** ^a^									
Total body fat (%)										
Baseline	32.0 ± 6.1	28.0 ± 9.5	30.9 ± 8.6	30.3 ± 6.6	0.262	0.024	**0.032**	0.084	0.428	0.012
End	31.0 ± 5.7	28.4 ± 9.9	29.8 ± 8.7	29.8 ± 6.0						
Trunk body fat (%)										
Baseline	37.7 ± 6.9	34.3 ± 9.9	37.7 ± 8.2	37.3 ± 6.8	0.156	0.038	**0.023**	0.094	0.856	0.001
End	36.6 ± 6.3	34.8 ± 10.3	35.9 ± 8.4	36.7 ± 5.8						
Upper limb muscles (kg)										
Baseline	4.6 ± 1.4	5.5 ± 1.3	5.3 ± 1.1	5.2 ± 1.3	0.968	0.000	0.428	0.012	0.088	0.054
End	4.9 ± 1.5	5.5 ± 1.3	5.4 ± 1.2	5.8 ± 1.2						
Thigh muscles (kg)										
Baseline	14.5 ± 3.6	16.5 ± 3.8	16.0 ± 3.2	16.9 ± 3.5	0.445	0.011	0.144	0.040	0.164	0.036
End	14.8 ± 3.8	16.3 ± 3.9	15.9 ± 3.2	16.8 ± 3.2						
Trunk muscles (kg)										
Baseline	21.4 ± 4.3	22.9 ± 4.3	22.7 ± 3.8	24.1 ± 3.7	0.523	0.008	0.442	0.011	0.479	0.010
End	21.4 ± 4.4	23.0 ± 4.5	23.1 ± 3.8	24.1 ± 3.8						
Body muscles (kg)										
Baseline	44.2 ± 9.4	48.7 ± 9.7	47.9 ± 8.4	50.4 ± 8.5	0.758	0.002	0.134	0.042	0.505	0.008
End	44.7 ± 10.0	48.6 ± 10.1	48.3 ± 8.5	50.6 ± 8.3						
**Bone health** ^a^										
Total BMD (g/cm^2^)										
Baseline	1.15 ± 0.09	1.18 ± 0.11	1.17 ± 0.08	1.19 ± 0.08	0.796	0.001	0.597	0.005	0.539	0.007
End	1.14 ± 0.08	1.17 ± 0.11	1.16 ± 0.09	1.19 ± 0.07						
Total BMC (g)										
Baseline	2,532.1 ± 448.1	2,695.3 ± 505.3	2,723.2 ± 390.8	2,716.5 ± 363.7	**0.014**	0.109	0.062	0.064	0.485	0.009
End	2,516.3 ± 425.5	2,700.6 ± 485.6	2,650.6 ± 362.5	2,689.6 ± 343.6						
Trunk BMD (g/cm^2^)										
Baseline	0.96 ± 0.09	0.96 ± 0.10	0.99 ± 0.12	0.99 ± 0.12	0.523	0.008	0.087	0.054	0.160	0.037
End	0.96 ± 0.09	0.95 ± 0.09	0.96 ± 0.09	0.99 ± 0.09						
Trunk BMC (g)										
Baseline	828.7 ± 158.6	820.7 ± 164.6	872.9 ± 137.6	867.5 ± 150.8	**0.046**	0.073	0.060	0.065	0.912	0.000
End	812.3 ± 151.8	832.5 ± 152.3	823.2 ± 131.1	849.4 ± 122.4						
Spine BMD (g/cm^2^)										
Baseline	1.09 ± 0.13	1.08 ± 0.14	1.14 ± 0.15	1.16 ± 0.14	**0.046**	0.073	0.826	0.001	0.234	0.027
End	1.11 ± 0.12	1.07 ± 0.13	1.09 ± 0.11	1.13 ± 0.15						
**Physical performance**										
Response latency (s)										
Baseline	675.3 ± 114.2	707.1 ± 91.5	689.6 ± 69.8	694.3 ± 187.9	0.663	0.004	0.589	0.005	0.357	0.016
End	603.7 ± 105.6	670.4 ± 116	650.4 ± 89.5	631.5 ± 101.9						
Single leg balance test (s)										
Baseline	11.4 ± 13.5	11.1 ± 7.9	12.9 ± 9.7	6.6 ± 3.6	0.729	0.002	0.163	0.036	0.353	0.016
End	18.1 ± 19.1	12.8 ± 14.3	17.5 ± 17.1	8.6 ± 5.9						
Vital capacity (ml)										
Baseline	2,647.7 ± 707.6	2,899.4 ± 879.4	3,014.9 ± 838.7	2,989.6 ± 766	0.385	0.014	0.688	0.003	0.993	0.000
End	2,671.8 ± 751.5	2,984.6 ± 864.5	3,170.1 ± 811.2	3,203.5 ± 714.6						
Grip strength (kg)										
Baseline	56.9 ± 20.4	59.9 ± 14.9	55.9 ± 19.7	57.9 ± 17	0.734	0.002	0.643	0.004	0.687	0.003
End	59.0 ± 19.0	62.2 ± 16.5	57.9 ± 14.8	62.0 ± 17.1						

a Data are mean ± standard deviation. VEG, vitamin D combined exercise intervention group; VDG, vitamin D intervention group; EG, exercise intervention group; CG, control group; Data were analyzed by a factorial repeated measures ANOVA, n = 14 in CG, were included. Boldface indicates statistically significance.

Body weight (mean = −1.1, 95%CI −1.9, −0.2), total BF% (mean = −1.1, 95%CI −2.0, −0.2), trunk BF% (mean = −1.8, 95%CI −2.9, −0.7), total BMC (mean = −73, 95%CI −118, −27) and trunk BMC (mean = −50, 95%CI −94, −6) were significantly decreased in EG, while they were not identified in VEG (Supplementary Table S2).

## Discussion

Our study found that in the middle-aged patients with T2DM 1) vitamin D supplementation for 12 weeks significantly increased 25(OH)D concentrations; 2) although there were no additive effect of vitamin D on bone health, body composition and physical performance beyond endurance exercise, vitamin D supplementation had a significant effect on bone health, and exercise was mainly on body fat percentage; 3) moreover, exercise only may have adverse effects on bone health, which was alleviated when combined with vitamin D supplementation.

Our previous studies reported that endurance exercise could increase serum 25(OH)D levels in short- or long-term interventions among young or older adults (Sun et al., 2017; Sun et al., 2018). Together with the evidence in mice that exercise significantly increased VDR expression (Makanae et al., 2015; Aly et al., 2016), we speculated that exercise combined with vitamin D supplementation intervention may have a additive effect on the improvement of musculoskeletal function via VDR pathway. However, our study had found no interactive effect of vitamin D and exercise on muscle mass and physical performance. According to our knowledge, few studies evaluated the combined effects in patients with T2DM, but there are several studies conducted on older adults. Our results was consistent with those in 148 older adults with combined interventions with light-moderate exercise training (Aoki et al., 2018); and inconsistent with those involved in resistance exercise while there were no control group setting (Antoniak and Greig, 2017). A recent clinical trial reported that weight-bearing exercise was more effective in improving muscle function than non-weight bearing endurance exercise (Villareal et al., 2017). In this study, exercise training was non-weight bearing, moderate-intensity, and this may be why exercise training did not have any main or additive effect beyond vitamin D on physical performance. Another possible reason maybe that patients in our study were younger and had better physical condition, in which the intervention effect was harder to achieve. Concurrent impacts of EX + VD on body composition and physical performance in patients with T2DM warrant further study in large-scale randomized controlled trials.

Diabetes cause damage to the microcirculation, which impairs the functioning of various organs and tissues, e.g., muscle tissues, kidney, eyes and nervous system. Compared with non-diabetic adults, adults with diabetes are at increased risk for physical disability, frailty and falling associated with progressive declines in leg muscle mass and strength (Nomura et al., 2018; Trierweiler et al., 2018). Progressive endurance exercise is typically one of the most important management strategies for T2DM prevention and control, while bone loss reduction accompanying with reduced weight significantly increased the risk of future fractures in patients with T2DM (Shah et al., 2011; Lipkin et al., 2014). In this study, after endurance exercise training, accompanying with the reduction of body fat percentage and decreased body weight, the loss of total and trunk BMCs were also observed in exercise only group, while they were alleviated in exercise combined vitamin D group. Vitamin D has the conventional benefit of maintaining bone health and reduces osteoporosis risk (Holick, 2007). Our results were consistently with those in the most recent study that vitamin D analogues supplementation increased bone health and levels of bone formation markers (e.g., osteocalcin) in patients with impaired glucose tolerance. Together these evidences suggest a combination of vitamin D and endurance exercise intervention while managing T2DM may help mitigate the adverse effects of weight loss by exercise on bone health.

In addition to classical effect of vitamin D on bone health, vitamin D receptors (VDRs) have been identified in several tissues, including skeletal muscle. Previous studies have consistently demonstrated that supplemental vitamin D at daily doses of ≥800 IU have beneficial effects on balance and muscle strength in frail elderly patients (≥60 years) (Muir and Montero-Odasso, 2011; Halfon et al., 2015). However, the results of this matter are more complex in young adults. Oosterwerff et al. reported that daily vitamin D supplementation at doses of 1200 IU for 4 months had no beneficial effects on the physical performance or physical activity among overweight subjects aged between 20 and 65 years (Oosterwerff et al., 2014). Inconsistently, a randomized, controlled trial showed that high doses of vitamin D (60,000 IU/week for 8 weeks followed by 60,000 IU/month for 4 months) combined with calcium supplementation significantly increased handgrip strength and walking distance in vitamin D-deficient Asian-Indians with a mean age of 31.5 ± 5.0 years (Gupta et al., 2010). In this study, supplementing 1,000 IU of vitamin D daily for 12 weeks had no positive effects on muscle strength and physical performance in patients with T2DM. These complex results may be due to different study settings. The patients with T2DM in this study were relatively younger (mean age, 50.0 ± 7.6 years that is, <60 years) and had a better physical status (muscle mass, 47.6 ± 8.9 kg; and grip strength, 28.9 ± 8.7 kg) than frail elderly patients.

The strength of this study includes a factorial randomized controlled trial design with high adherence to a 12-week intervention. Our study provides a novel finding that vitamin D combined with exercise training may mitigate the potential adverse effects of exercise on bone health while managing T2DM. This study has some limitations. First, the study duration was relatively short, and it is unclear whether long-term intervention studies support our findings. Second, the outcome reported in this study was a secondary outcome of a registered randomized controlled clinical trial. Third, subjects included in the study were middle-aged and without co-supplements, thus caution is needed while interpreting results. Finally, the sample size and effect size was relatively small, although a post hoc power calculation would still give us a at least 90% chance to demonstrate the interactive effect on bone mass content, which was defined as statistically significant at *p* < 0.05 at the end points. Hence, larger and long-term studies on the effects of vitamin D supplementation or exercise training intervention on bone health and physical performance are encouraged.

## Conclusion

In summary, vitamin D supplementation at a dose of 1,000 IU/day over 12 weeks significantly alleviated vitamin D deficiency and played an important role in maintaining bone health. Our findings provide useful information regarding the management of patients with T2DM and explore the potential effects of vitamin D supplementation combined with exercise training. Further research is needed to evaluate the long-term effects of vitamin D supplementation combined with exercise on patients with T2DM and understand the mechanism of this association.

## Data Availability

The original contributions presented in the study are included in the article (Supplementary Material), further inquiries can be directed to the corresponding author.
