# Supplementary material for: Does vitamin D supplementation improve bone health, body composition and physical performance beyond endurance exercise in patients with type 2 diabetes: A secondary analysis of randomized controlled trial
Source: Front Physiol. 2022 Sep 28;13:1003572. doi: 10.3389/fphys.2022.1003572 (PMC9554259; doi:10.3389/fphys.2022.1003572)
Supplement: Supplementary file 1 [file Table1.docx]

**Supplemental Table 1 The protocol of endurance exercise training**

| **Stage** | **HRmax (%)** | **RPE** | **Time** |
| --- | --- | --- | --- |
| Week 1 | 65-70% | 11-13 | 60 min (including 5 min warm up, and 5 min recovery exercise) |
| Week 2-4 | 70-75% | 11-13 | 60 min (including 5 min warm up, and 5 min recovery exercise) |
| Week 5-8 | 75-80% | 12-13 | 60 min (including 5 min warm up, and 5 min recovery exercise) |
| Week 9-12 | 80% | 13 | 60 min (including 5 min warm up, and 5 min recovery exercise) |

Note: HRmax, Maximum heart rate; RPE, Rating of perceived exertion.

**Supplemental Table 2** Mean differences (95% CI) of bone health, body composition and physical performance variables from the endpoint with each group

| Variables | VEG | VDG | EG | CG | P | | |
| --- | --- | --- | --- | --- | --- | --- | --- |
|  | N=15 | N=14 | N=14 | N=14 | P_vitamin D_ | P_exercise_ | P_interaction_ |
| **Body composition** |  |  |  |  |  |  |  |
| Δ Body weight ^a^ | -0.3 (-1.3, 0.7) | -0.3 (-1.6, 1.0) | **-1.1 (-1.9, -0.2)** | -0.2 (-1.1, 0.7) | 0.516 | 0.386 | 0.341 |
| Δ BMI ^a^ | 0.7 (-1.0, 2.5) | -0.1 (-0.6, 0.4) | -0.3 (-0.6, 0.0) | -0.1 (-0.4, 0.3) | 0.245 | 0.522 | 0.211 |
| Δ Total body fat (%) | -1.0 (-2.1, 0.2) | 0.4 (-0.7, 1.6) | **-1.1 (-2.0, -0.2)** | -0.5 (-1.2, 0.2) | 0.262 | **0.032** | 0.428 |
| Δ Trunk body fat (%) | -1.1 (-2.6, 0.5) | 0.4 (-1.0, 1.9) | **-1.8 (-2.9, -0.7)** | -0.5 (-1.6, 0.6) | 0.156 | **0.023** | 0.856 |
| Δ Body muscles (kg) | 0.5 (-0.2, 1.3) | -0.2 (-0.8, 0.4) | 0.4 (-0.4, 1.2) | 0.1 (-0.4, 0.7) | 0.758 | 0.134 | 0.505 |
| Δ Trunk muscles (kg) | 0.0 (-0.7, 0.8) | 0.0 (-0.6, 0.6) | 0.4 (-0.0, 0.9) | -0.1 (-0.3, 0.2) | 0.523 | 0.442 | 0.479 |
| Δ Upper limb muscles (kg) | 0.2 (0.0, 0.5) | 0.0 (-0.1, 0.2) | 0.1 (-0.1, 0.2) | 0.2 (0.0, 0.3) | 0.968 | 0.428 | 0.088 |
| Δ Thigh muscles (kg) | 0.3 (-0.1, 0.6) | -0.2 (-0.4, 0.0) | -0.1 (-0.6, 0.4) | -0.1 (-0.3, 0.2) | 0.445 | 0.144 | 0.164 |
| **Bone health** |  |  |  |  |  |  |  |
| Δ Total BMD (g/cm^2^) | -0.01 (-0.02, 0.01) | -0.00 (-0.01, 0.00) | -0.00 (-0.01, 0.01) | -0.01 (-0.01, 0.01) | 0.796 | 0.597 | 0.539 |
| Δ Total BMC (g) | -16 (-41, 9) | 9 (-37, 55) | **-73 (-118, -27)** | -29 (-71, 13) | **0.014** | 0.062 | 0.485 |
| Δ Trunk BMD (g/cm^2^) | -0.01 (-0.01, 0.00) | -0.00 (-0.01, 0.01) | -0.03 (-0.08, 0.01) | 0.01 (-0.02, 0.04) | 0.523 | 0.087 | 0.160 |
| Δ Trunk BMC (g) | -16 (-38, 5) | 16 (-19, 52) | **-50 (-94, -6)** | -14 (-51, 22) | **0.046** | 0.600 | 0.912 |
| Δ Spine BMD (g/cm^2^) | 0.01 (-0.01, 0.04) | -0.01 (-0.03, 0.00) | -0.05 (-0.11, 0.02) | -0.03 (-0.07, 0.02) | **0.046** | 0.826 | 0.234 |
| **Physical performance** |  |  |  |  |  |  |  |
| Δ Grip strength (kg) ^a^ | 1.1 (-1.1, 3.2) | 1.1 (-1.8, 4.1) | 1.0 (-1.9, 3.9) | 2.0 (-0.3, 4.4) | 0.734 | 0.643 | 0.687 |
| Δ Response latency (s) ^a^ | **-71.7 (-111.0, -32.3)** | -36.7 (-104.2, 30.6) | **-39.3 (-74.1, -4.5)** | -62.8 (-138.8, 13.2) | 0.598 | 0.519 | 0.284 |
| Δ Single leg balance test (s) ^a^ | 39.4 (-18.2, 97.0) | 14.4 (0.6, 28.3) | 39.2 (-2.6, 81.0) | 10.6 (4.2, 17.0) | 0.475 | 0.878 | 0.417 |
| Δ Vital capacity (mL) ^a^ | 24.1 (-365.7, 413.8) | 85.2 (-279.5, 444.9) | 155.3 (-51.4, 361.9) | 213.9 (-61, 488.7) | 0.385 | 0.688 | 0.993 |

Data are mean (95% confidence interval). Δ, changes from the endpoint to the pre. VEG, vitamin D combined exercise intervention group; VDG, vitamin D intervention group; EG, exercise intervention group; CG, control group; BMI, body mass index; BMD, bone mineral density; BMC, bone mineral content. Data were analyzed by mixed factorial analyses of variance. ^a^, n=15 in CG were included.
